# PET in conjunction with resting-state functional MRI for the study of chronic disorders of consciousness

**DOI:** 10.1093/braincomms/fcaf495

**Published:** 2025-12-23

**Authors:** Alice Deruti, Jean P Medina Carrion, Mario Stanziano, Ludovico D'Incerti, Davide Sattin, Davide Rossi Sebastiano, Stefania Ferraro, Francesca G Magnani, Greta Demichelis, Michela Picchetti, Riccardo Benti, Marina Grisoli, Matilde Leonardi, Maria G Bruzzone, Giorgio Marotta, Cristina Rosazza, Anna Nigri

**Affiliations:** Neuroradiology Unit, Diagnostic and Technology Department, Fondazione IRCCS Istituto Neurologico Carlo Besta, Milan 20133, Italy; Neuroradiology Unit, Diagnostic and Technology Department, Fondazione IRCCS Istituto Neurologico Carlo Besta, Milan 20133, Italy; Neuroradiology Unit, Diagnostic and Technology Department, Fondazione IRCCS Istituto Neurologico Carlo Besta, Milan 20133, Italy; Neurosciences Department ‘Rita Levi Montalcini’, University of Turin, Turin 10126, Italy; Neuroradiology Unit, Diagnostic and Technology Department, Fondazione IRCCS Istituto Neurologico Carlo Besta, Milan 20133, Italy; Neuroradiology Unit, Children’s Hospital A. Meyer-University of Florence, Florence 50139, Italy; IRCCS Istituti Clinici Scientifici Maugeri of Milan, Milan 20138, Italy; Neurophysiopathology Unit, Department of Diagnostic and Technology, Fondazione IRCCS Istituto Neurologico Carlo Besta, Milan 20133, Italy; Neuroradiology Unit, Diagnostic and Technology Department, Fondazione IRCCS Istituto Neurologico Carlo Besta, Milan 20133, Italy; MOE Key Laboratory for Neuroinformation, School of Life Science and Technology, University of Electronic Science and Technology of China, Chengdu 611731, China; Neurology, Public Health, Disability Unit, Fondazione IRCCS Istituto Neurologico Carlo Besta, Milan 20133, Italy; Neuroradiology Unit, Diagnostic and Technology Department, Fondazione IRCCS Istituto Neurologico Carlo Besta, Milan 20133, Italy; Neuroradiology Unit, Diagnostic and Technology Department, Fondazione IRCCS Istituto Neurologico Carlo Besta, Milan 20133, Italy; Nuclear Medicine Unit, Fondazione IRCCS Ca’ Granda Ospedale Maggiore Policlinico, Milan 20122, Italy; Neuroradiology Unit, Diagnostic and Technology Department, Fondazione IRCCS Istituto Neurologico Carlo Besta, Milan 20133, Italy; Neurology, Public Health, Disability Unit, Fondazione IRCCS Istituto Neurologico Carlo Besta, Milan 20133, Italy; Neuroradiology Unit, Diagnostic and Technology Department, Fondazione IRCCS Istituto Neurologico Carlo Besta, Milan 20133, Italy; Nuclear Medicine Unit, Fondazione IRCCS Ca’ Granda Ospedale Maggiore Policlinico, Milan 20122, Italy; Department of Clinical Sciences and Community Health, University of Milan, Milan 20122, Italy; Neuroradiology Unit, Diagnostic and Technology Department, Fondazione IRCCS Istituto Neurologico Carlo Besta, Milan 20133, Italy; Department of Humanistic Studies, University of Urbino Carlo Bo, Urbino 61029, Italy; Neuroradiology Unit, Diagnostic and Technology Department, Fondazione IRCCS Istituto Neurologico Carlo Besta, Milan 20133, Italy

**Keywords:** etiology, FDG-PET, minimally conscious state, resting-state fMRI, vegetative state/unresponsive waking state

## Abstract

In Disorders of Consciousness, ^18^F-fluorodeoxyglucose PET (FDG-PET) is known to be effective in distinguishing vegetative state/unresponsive wakefulness syndrome from minimally conscious state, and when combined with MRI techniques, the risk of misdiagnosis decreases. However, FDG-PET studies on chronic patients with different etiologies (traumatic, vascular, and anoxic brain injury) are limited, and the association between metabolic activity and resting-state functional MRI (fMRI) networks remains unclear. This study combined FDG-PET with resting-state functional MRI and MRI to assess: i) the diagnostic accuracy of FDG-PET metabolism in different etiological groups of patients; ii) whether resting-state fMRI networks presence or absence was associated with higher versus lower FDG-PET metabolism. A group of 84 chronic patients underwent FDG-PET (47 vegetative state/unresponsive wakefulness syndrome, 31 minimally conscious state, and six emerged from a minimally conscious state), equally distributed in traumatic, vascular, and anoxic etiologies. Eight cases of covert cortical processing were identified. A subgroup of 68 patients also underwent resting-state fMRI. Standardized uptake values were calculated for these areas of interest: 10 resting-state fMRI networks, the precuneus, and a whole-brain mask. Patients in a vegetative state/unresponsive wakefulness syndrome exhibited a significant decrease in metabolism compared to patients in a minimally conscious state across all areas of interest. Patients with covert cortical processing showed intermediate metabolic levels between the two diagnostic categories. The anoxic group displayed a severe decrease in metabolism compared to patients with traumatic and vascular etiologies. The highest diagnostic accuracy among the areas of interest was reached in the precuneus and medial visual network (Area Under the Curve, AUC = 0.82–0.83). However, when anoxic patients were excluded, the diagnostic accuracy did not reach statistical significance, although the medial visual network and precuneus retained a trend of gradually increasing metabolism as clinical conditions improved. Identification of resting-state functional MRI networks was associated with increased metabolism in all networks at the group level, even excluding patients with severe structural damage. FDG-PET proves to be a technique capable of distinguishing vegetative state/unresponsive wakefulness syndrome from minimally conscious state even in chronic patients, although its diagnostic accuracy can be significantly affected by the etiology. There is a concordance between the metabolism level and the presence of resting-state fMRI networks.

## Teaser

Deruti *et al*. report that PET effectively differentiates vegetative state from minimally conscious state even in chronic patients, although its diagnostic accuracy can be significantly affected by the etiology. It also shows that the presence of resting-state functional MRI networks is associated with better metabolism levels.

## Graphical Abstract

**Graphical Abstract:**
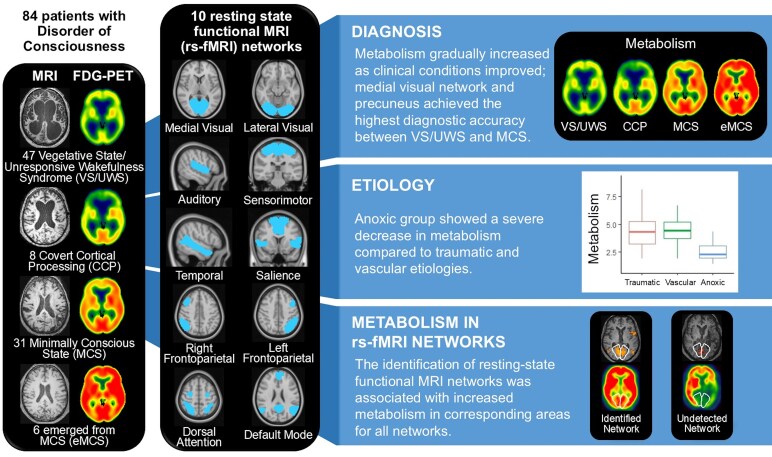

## Introduction

Disorders of Consciousness (DOC) encompass conditions of profound disruption in arousal and/or awareness due to severe brain damage,^1^ spanning from coma, vegetative state/unresponsive wakefulness syndrome (VS/UWS, characterized by arousal without behavioural signs of awareness), to minimally conscious state (MCS, marked by fluctuating awareness and appropriate responses to stimuli).

Neuroimaging techniques respond to a clinical need for more accurate diagnosis and prognosis, providing insights into brain structure and function. Among the functional techniques, positron emission tomography with 18F-fluorodeoxyglucose (FDG-PET) was one of the first methods used to quantify cerebral glucose metabolism: patients with DOC demonstrated a marked decrease in brain metabolism, up to 50% of normal values.^1,2^ Current evidence underscores FDG-PET's high sensitivity and specificity in distinguishing between VS/UWS and MCS,^3^ achieving an area under the curve (AUC) of 0.90.^4^ The precuneus, with fronto-parietal and visual regions, shows the best metabolic discriminative ability.^3,4^ Indeed, the European guidelines recommended performing FDG-PET as part of the multimodal assessment in patients in VS/UWS.^5^

Resting-state functional MRI (rs-fMRI) is another functional technique applied to DOC to examine the integrity of neuronal activity patterns across specific brain regions, forming rs-fMRI brain networks.^6,7^ The functional connectivity of some networks, such as default mode network and visual networks, has proven to be important in discriminating between patients in VS/UWS and in MCS,^8-11^ and a higher number of networks is associated with a better clinical condition.^10,12,13^ The use of functional imaging techniques has led to the identification of new diagnostic categories, such as covert cortical processing (CCP), which is characterized by intact associative cortical responses to environmental stimuli despite the absence of behavioural evidence of stimulus processing.^14^

Moreover, FDG-PET and rs-fMRI generally display good agreement: the former measures brain glucose consumption, while the latter detects changes in cerebral blood oxygenation, both reflecting neuronal activity.^15,16^ Indeed, a significant correlation between FDG-PET and rs-fMRI-based maps has been observed in 11 VS/UWS and 4 locked-in syndrome patients, showing high similarity between rs-fMRI and FDG-PET metabolic maps.^17^

However, while the diagnostic accuracy of FDG-PET has been reported for anatomical regions, there is a lack of assessment of metabolism in functional areas corresponding to resting-state networks, an approach widely used in patients with DOC. Additionally, although a voxel-level correlation between the two techniques has been demonstrated,^17^ it remains unclear whether the presence of specific networks corresponds to higher metabolic levels in the associated regions.

Furthermore, patients with DOC display high clinical heterogeneity associated with the etiology. Traumatic patients, generally younger, show variable brain lesions depending on the extent and site of the trauma; vascular patients typically show an asymmetric pattern of lesions with the worst premorbid comorbidities; anoxic patients exhibit diffuse brain alterations and global metabolism decreases with higher clinical complexity.^18-21^ Most FDG-PET studies reported a single diagnostic accuracy value without distinguishing between different etiologies or between acute and chronic conditions.^3,4,22^ Therefore, the reduced brain metabolism observed in anoxic patients, combined with the predominance of VS/UWS cases among them, may affect the diagnostic accuracy of FDG-PET.

Hence, we aim to characterize the brain metabolism of 84 chronic patients with DOC and patients who emerged from MCS (eMCS) with different etiologies, namely traumatic, vascular, and anoxic. In addition, we aim to identify potential cases of CCP and to evaluate their metabolic patterns. The diagnostic accuracy of FDG-PET for distinguishing between VS/UWS and MCS was assessed throughout the entire group, as well as in the different etiologies. In addition, FDG-PET was combined with rs-fMRI: we evaluated the FDG-PET metabolism of patients in 10 rs-fMRI networks and we determined whether higher or lower levels of metabolism were associated with the presence versus absence of resting-state fMRI networks, also considering structural MRI data.

## Material and methods

### Participants

A group of chronic patients with DOC was prospectively enrolled in a 1-week program of multidisciplinary assessment during 2011–2013 at the Coma Research Center of the Fondazione IRCCS Istituto Neurologico ‘Carlo Besta’ in Milan, Italy.^10,23,24^ All patients performing FDG-PET at the Fondazione IRCCS Ca’ Granda Ospedale Maggiore Policlinico, Milan, Italy, were included (*N* = 86); this sample overlaps (*N* = 83) with that one reported in Rosazza et al..^9^ Two patients were excluded from the analyses (see *Statistical analyses*). The final sample of 84 patients was clinically classified into 47 VS/UWS, 31 MCS, and six eMCS, with a median disease duration of 30 months (range of 3–252). The diagnoses were made according to the Aspen criteria^25^ and the Coma Recovery Scale-revised (CRS-R),^26^ by adopting the Italian version of the CRS-R.^27^ CRS-R modified score was also considered for correlations.^28^ Regarding the etiology, 24 patients had a traumatic brain injury, 31 had a vascular brain injury, and 29 had an anoxic brain injury, including hypoxic-ischemic and anoxic-ischemic, as well as cardiac respiratory arrest. A subsample of 68 patients, consisting of 38 VS/UWS, 25 MCS, and five eMCS, also underwent rs-fMRI acquisition, on average within 2.6 days of the FDG-PET. No participants received sedatives during imaging acquisition, as confirmed by the researcher (AN, CR, or MGB) who was present during the scanning session. In Table 1, patients’ demographic and clinical information are reported. The institutional ethics committee of Istituto ‘Carlo Besta’ approved the study, which was conducted in accordance with the Declaration of Helsinki, and patients’ legally authorized representatives provided their signed informed consent. Cases suggestive of diagnostic changes, with respect to the admission diagnosis, were shared with the clinical team responsible for the patient's care.

**Table 1:** Summary of demographic and clinical variables

Diagnosis	*N*	Age, yr	Sex, M/F	Traumatic	Vascular	Anoxic	Disease Duration, mo	CRS-R
FDG-PET								
All patients	84	51 (19–83)	47/37	24	31	29	30 (3–252)	7 (5–22)
VS/UWS	47	52 (23–76)	30/17	10	13	24	25 (5–252)	7 (5–8)
MCS	31	47 (19–83)	13/18	12	16	3	39 (6–209)	10 (7–16)
eMCS	6	52 (19–66)	4/2	2	2	2	14 (3–31)	18 (14–22)
FDG-PET and rs-fMRI								
All patients	68	49 (19–83)	39/29	18	26	24	29 (5–252)	7 (5–21)
VS/UWS	38	49 (23–79)	28/10	9	10	19	25 (5–252)	7 (5–8)
MCS	25	47 (19–83)	8/17	8	14	3	37 (6–209)	10 (7–16)
eMCS	5	54 (37–66)	3/2	1	2	2	15 (9–31)	18 (14–21)

Age, disease duration, and CRS-R total scores are reported as median values. Values in parentheses indicate minimum and maximum values. *N*, number; yr, year; M, male; F, female; mo, months.

A subsample of patients in VS/UWS and MCS− who had undergone visual task-based fMRI^29^ and auditory language paradigms^30^ was considered to identify potential cases of CCP^14^ (see Supplementary Materials and Supplementary Table 1).

### FDG-PET

For static FDG-PET data, scanning acquisition (with head holder and pillows to restrain head) and pre-processing procedures are detailed in Rosazza *et al*..^9^ Briefly, for each subject, standardized uptake value (SUV) maps were coregistered to individual 3D T1-weighted images, spatially normalized to MNI space and smoothed with a 10 mm isotropic Gaussian filter.^9^

### MRI data acquisition and rs-fMRI processing

The MRI protocol included gradient echo-planar images (EPI) for rs-fMRI and 3D T1-weighted images, as reported in Medina *et al*..^10,13^ The following 10 resting-state networks were considered: medial visual (MVIS), lateral visual (LVIS), auditory (AUD), sensorimotor (SM), temporal (TEMP), salience (SAL), right frontoparietal (R-FP), left frontoparietal (L-FP), dorsal attention (DAN), and default mode (DMN) networks. For each network, a template was obtained from healthy participants using Independent Component Analysis (Melodic, FSL tool^31^), as described in Medina *et al*..^10,13^

To obtain an assessment of the degree of anatomical damage, a qualitative MRI rating approach was used for its feasibility across all patients, given the severe structural alterations.^20^ As reported in Rosazza *et al*.^9,^ two expert neuroradiologists rated the severity of anatomical and signal abnormality at network node locations according to the following scale: 0 (severely damaged), 1 (recognizable but distorted morphology), 2 (moderate anatomical damage), 3 (mild anatomical damage), and 4 (normal-appearing). The scores were then averaged together, with inter-rater agreement at *ρ* = 0.88.

The seed-based analysis on rs-fMRI data was performed with DPARSF-A^32^ using 40 seeds (6 mm radius) for the 10 networks. The choice of seed location for each network was performed according to Medina *et al*.^13^, following previous literature coordinates for DAN,^33^ SAL,^8,34^ L-FP,^35^ R-FP,^35^ TEMP,^36^ and DMN^9^ and for the remaining networks (MVIS, LVIS, AUD, and SM).^6,8,10,37^ To reduce noise and enhance spatial specificity of seed-based connectivity maps: i) positive z-score maps were considered; ii) maps from seeds included in nodes with MRI ratings ≤ 1 were excluded in order to avoid damaged areas; iii) for each network, the maps were merged considering the median value for each voxel; iv) a network-specific threshold was applied (network mean value plus 2 standard deviations); v) clusters with less than 150 voxels were removed.

Based on the evaluation by two rs-fMRI experts, a network was classified as ‘*Identified network*’ if the patient's connectivity map exhibited clusters of correlated activity forming a clear and well-defined functional pattern. In the absence of such features, it was classified as ‘*Undetected network*’.

Neuroradiologists and rs-fMRI experts were blinded to all patient clinical data.

### Areas of interest

**Figure 1:**
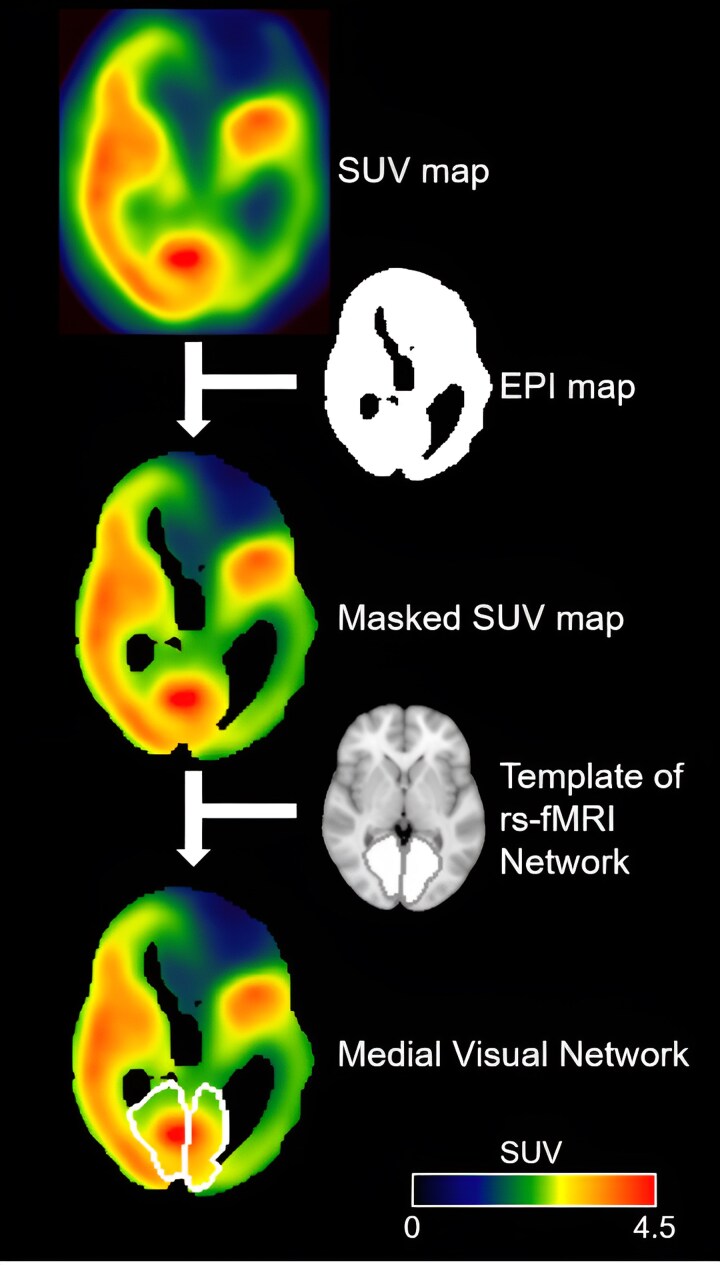
**Workflow of the procedure applied to FDG-PET data of patients with DOC.** The SUV map was masked using the EPI map to exclude areas of cerebrospinal fluid and anatomical damage. Then, a template for each area of interest (networks, precuneus, and whole brain) was used on the masked SUV map to extract SUV values. For illustrative purposes, a single subject is presented, and the MVIS template was superimposed. EPI, echoplanar images; SUV, standardized uptake values.

To assess FDG-PET in areas of interest derived from resting-state fMRI, the SUV map was constrained to neural areas using a whole-brain mask derived from each patient’s EPI images (Fig. 1). EPI images were thresholded within the 20th–75th percentile range of the BOLD signal percentile range to exclude cerebrospinal fluid and damage. This allows us to obtain an SUV value that is representative of the residual metabolism of the area of interest, even for patients with extensive and/or lateralized damage.

In addition to the 10 networks, the precuneus and the whole brain were included as areas of interest, where the precuneus, known to be a significant hub, was derived from the DMN by selecting the posterior node^9^ and the whole brain was used as reference. To account for subject-specific anatomical variability in the spatial configuration of rs-fMRI networks, the templates were expanded using a spherical kernel with a radius of 10 mm. For each area of interest, the SUV value was extracted as the median value of all included voxels (Fig. 1).

### Statistical analyses

To assess residual metabolism in patients with DOC, the FDG-PET analyses were conducted on the entire sample, stratified by etiology, and also by grouping traumatic and vascular cases together, excluding anoxic patients due to the greater severity of brain damage. Results were considered significant at *P* < 0.05, corrected for multiple comparisons using the Holm-Bonferroni method.

Two patients were excluded because they were classified as outliers for SUV values of the areas of interest using the DBscan algorithm (Density-Based Spatial Clustering of Applications with Noise) in the package dbscan in RStudio.

#### FDG-PET voxel-wise analyses

To highlight the main effect of each diagnostic category, an average map was obtained for patients in VS/UWS, CCP, MCS, and eMCS on SUV maps. Then, to assess significant metabolic differences between patients in VS/UWS and in MCS, a between-group analysis was performed using SPM12. In order to exclude areas in close proximity to the ventricles, we applied a mask obtained by thresholding the average SUV map across all patients at 40%. In addition, regression voxel-wise analyses were done on the entire sample using the CRS-R score and disease duration as regressors of interest.

#### Areas of interest analyses

For each area of interest, two separate Jonckheere-Terpstra trend tests were performed to assess the metabolic change as the clinical condition improved: the first comparing VS/UWS, MCS, and eMCS; the second comparing VS/UWS, CCP, and MCS, with CCP considered as an intermediate group.

A PERmutational Multivariate ANalysis of VAriance (PERMANOVA^38^) was first conducted across all areas of interest to assess the presence of a significant global effect between patients in VS/UWS and MCS, as well as between anoxic and non-anoxic patients in VS/UWS. Upon confirming global significance in either comparison, post-hoc analyses using Mann-Whitney tests were performed to evaluate differences between the respective groups for each area of interest. Receiver Operating Characteristic (ROC) curves were built on patients in VS/UWS and MCS using a 1000-repetition bootstrap for each area of interest, considering a balanced proportion by etiology between training and test samples. Both AUC and balanced accuracy (Bal ACCU) were calculated to assess the capability of SUV values in predicting the diagnosis. The misclassified cases, according to the AUC curve, in at least 75% of areas of interest were evaluated considering the number of rs-fMRI networks, where available, in order to understand if the misclassification was explained by another functional technique.

Finally, Spearman ρ correlations were performed to assess the association between SUV values in each area and clinical data (CRS-R score, CRS-R modified score, and disease duration).

#### Association of FDG-PET metabolism with rs-fMRI networks

To assess whether the presence or absence of rs-fMRI networks is associated with higher or lower levels of metabolism in patients with DOC, for each network, the whole sample (*N* = 68) was divided into two groups: patients with a recognizable network (*Identified network* group) and patients without a recognizable network (*Undetected network* group). To assess the difference between the two groups, the Mann-Whitney test was performed on the SUV values of each network. In addition, the comparison between the two groups was also performed by excluding patients whose MRI ratings were ≤1 in all seeds of the network.

## Results

### Voxel-wise results

**Figure 2:**
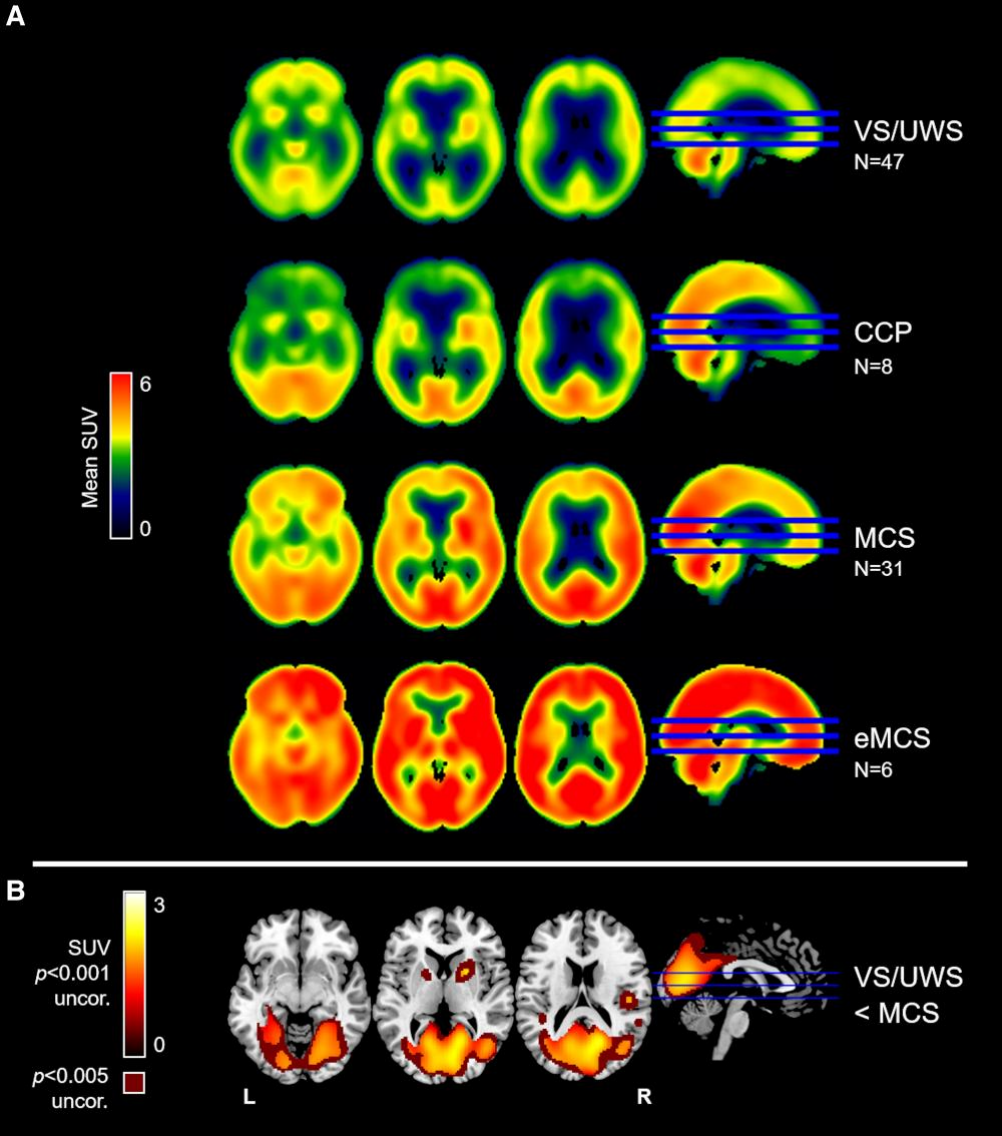
**FDG-PET metabolism observed in patients in VS/UWS, CCP, MCS, and eMCS, and the difference between patients in VS/UWS and in MCS.** (**A**) Average maps of SUV values by diagnosis in VS/UWS (*N* = 47), CCP (*N* = 8), MCS (*N* = 31), and eMCS (*N* = 6) categories. (**B**) Subtraction of the average SUV map of patients in MCS from that of patients in VS/UWS, restricted to voxels showing statistically significant differences at *P* < 0.001 uncorrected (red to yellow scale) and *P* < 0.005 uncorrected (dark red). The result was overlaid on an anatomical MNI template. The axial sections for (**A**) are z = −14, 2, 18 and for (**B**) are z = −8, 5, 18. L, left; R, right; uncor., uncorrected.

In our group of 84 patients (47 VS/UWS, 31 MCS, and six eMCS), the average SUV map of each diagnosis was reported in Fig. 2A. The VS/UWS group showed a widespread decrease of metabolism in cortical regions compared to patients in MCS and eMCS, while the activity in the brainstem and cerebellum was more preserved. The MCS group showed a decreased metabolism mainly in the frontal regions compared to patients in eMCS, while the posterior and cerebellar areas presented higher metabolic activity. An overall high metabolism was observed in patients in eMCS. The regression analyses across all patients using the CRS-R score and disease duration showed no significant results.

The MCS compared to the VS/UWS group showed a significantly higher metabolism in bilateral posterior areas, including the precuneus, posterior cingulate, cuneus, lingual, fusiform, occipital gyri, putamen, and the right globus pallidus (Fig. 2B, Supplementary Fig. 4 with T-scores values, Supplementary Table 2). In Fig. 2B, the subtraction of the average SUV map of patients in MCS from that of patients in VS/UWS was reported for comparison with previous studies.^3^

**Figure 3:**
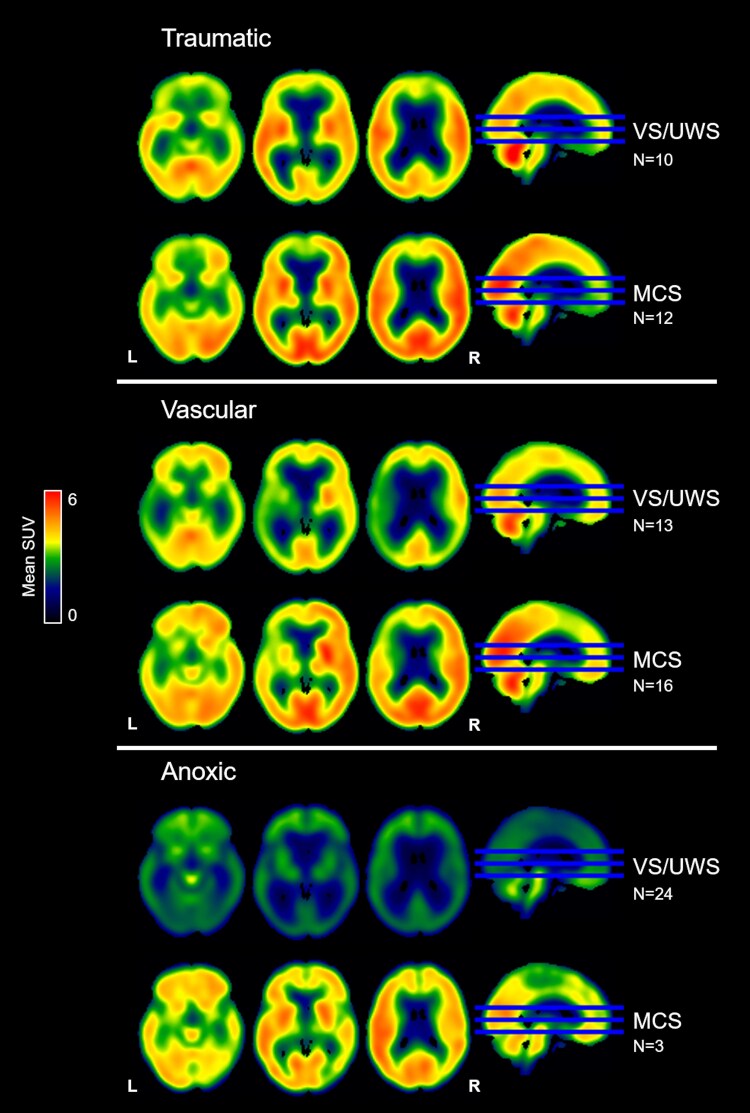
**FDG-PET metabolism observed in patients in VS/UWS and in MCS grouped by etiology: traumatic, vascular, and anoxic.** Average maps of SUV values by diagnosis for traumatic, vascular, and anoxic patients. The axial sections are z = −14, 2, 18. L, left; R, right.

For the traumatic and vascular subsample, the pattern of metabolism between VS/UWS and MCS was similar, while for the anoxic group, the VS/UWS showed a widespread massive decrease that was considerably lower than in MCS, although in this group, there were only three patients (Fig. 3).

Moreover, in our sample, eight CCP cases out of 28 patients (29%; Supplementary Table 1) showed fMRI task-related activations in associative areas and displayed an intermediate metabolic pattern between patients in VS/UWS and those in MCS, particularly involving the posterior regions (Fig. 2A).

Color-blind versions of Fig. 1 and 2A and 3 are available in Supplementary Materials (Supplementary Fig. 1 and 2, and 3). All maps were uploaded to NeuroVault (https://neurovault.org/collections/21725/).

### SUV in areas of interest

Considering the whole sample, the trend test among VS/UWS, MCS, and eMCS showed a significant gradual increase of metabolism in all the areas of interest as the clinical condition improved (*P* < 0.01; Table 2). Similarly, the trend test among VS/UWS, CCP, and MCS was significant in all area (*P* < 0.05).

**Table 2:** SUV values results obtained for each area of interest (networks, precuneus, and whole brain) in patients with DOC

Areas of Interest	Jonckheere-Terpstra trend test (*TJ, P-value*)	Mann-Whitney *U* test (*U, P-value*)	AUC [C.I.]	Bal ACCU	CRS-R (*ρ, P-value*)	CRS-R modified (*ρ, P-value*)
All Sample (N = 84)						
	VS/UWS < MCS < eMCS	VS/UWS versus MCS			VS/UWS MCS eMCS	
MVIS	1470, **0.001**	310, **<0.001**	**0.83** [0.61–0.97]	0.65	0.43, **<0.001**	0.43, **0.001**
LVIS	1393, **0.001**	385, **0.003**	**0.78** [0.55–0.96]	0.63	0.35, **0.011**	0.34, **0.021**
AUD	1380, **0.001**	427, **0.011**	0.74 [0.49–0.95]	0.60	0.32, **0.011**	0.32, **0.025**
SM	1328, **0.003**	457, **0.017**	0.74 [0.47–0.95]	0.60	0.26, **0.017**	0.26, **0.025**
TEMP	1349, **0.002**	451, **0.017**	0.73 [0.44–0.95]	0.60	0.32, **0.011**	0.32, **0.025**
SAL	1321, **0.003**	497, **0.036**	0.71 [0.44–0.95]	0.59	0.29, **0.016**	0.30, **0.025**
R-FP	1367, **0.001**	426, **0.011**	0.75 [0.48–0.97]	0.60	0.33, **0.011**	0.33, **0.023**
L-FP	1320, **0.003**	499, **0.036**	0.71 [0.41–0.95]	0.58	0.34, **0.011**	0.31, **0.025**
DAN	1385, **0.001**	400, **0.005**	**0.77** [0.52–0.96]	0.62	0.34, **0.011**	0.33, **0.021**
DMN	1429, **0.001**	382, **0.003**	**0.78** [0.53–0.96]	0.63	0.36, **0.007**	0.36, **0.014**
Precuneus	1490, **0.001**	313, **<0.001**	**0.82** [0.62–0.98]	0.66	0.44, **<0.001**	0.43, **0.001**
Whole Brain	1393, **0.001**	395, **0.004**	**0.78** [0.52–0.96]	0.62	0.34, **0.011**	0.35, **0.015**

The table shows the results of the whole sample (*N* = 84). The trend test and correlations of SUV values with the CRS-R and the modified CRS-R were calculated considering the three diagnoses (patients in VS/UWS, MCS, and eMCS). The Mann-Whitney test, AUC and Bal ACCU were obtained comparing patients in VS/UWS and in MCS. The results highlighted in bold indicate that the lower limit of the AUC confidence interval is higher than 0.5. All *P*-values are corrected for multiple comparisons using the Holm-Bonferroni method, and significant *P*-values are shown in bold. Confidence intervals are reported at 95%. C.I., confident interval.

**Figure 4:**
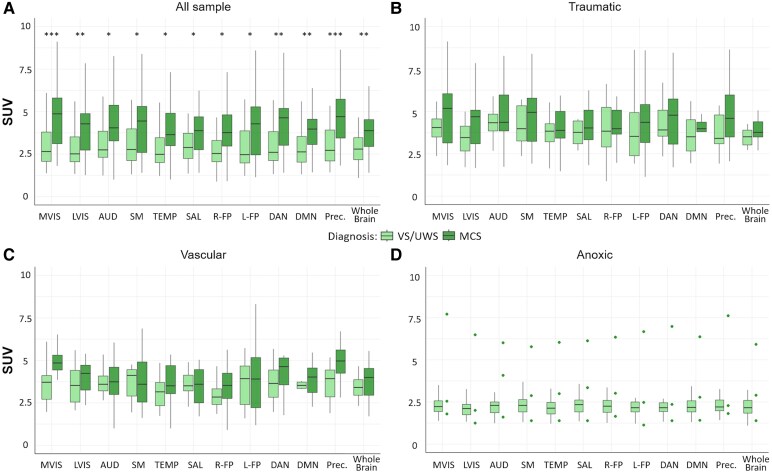
**SUV values of the areas of interest in the whole sample and in different etiologies of patients with DOC.** Boxplots of median SUV values in patients in VS/UWS and in MCS for each area of interest (networks, precuneus, and whole brain) are shown: (**A**) across the whole sample (VS/UWS = 47, MCS = 31); (**B**) the traumatic subgroup (VS/UWS = 10, MCS = 12); (**C**) in the vascular subgroup (VS/UWS = 13, MCS = 16); (**D**) in the anoxic subgroup (VS/UWS = 24, MCS = 3). In the anoxic subgroup, the patients in MCS are represented as dots since there were only three. For comparison (**A**), in which the PERMANOVA test yielded a significant global effect, differences between VS/UWS and MCS in each area of interest were evaluated using Mann-Whitney tests; significant *P*-values, corrected for multiple comparisons using the Holm-Bonferroni method, are indicated with asterisks. Significance levels: * *P* < 0.05, ** *P* < 0.01, *** *P* < 0.001, **** *P* < 0.0001. SUV, standardized uptake values; prec., precuneus; MVIS, medial visual; LVIS, lateral visual; AUD, auditory; SM, sensorimotor; TEMP, temporal; SAL, salience; R-FP, right frontoparietal; L-FP, left frontoparietal; DAN, dorsal attention; DMN, default mode networks.

As shown in Fig. 4A, metabolism was significantly lower in patients in VS/UWS than in MCS (PERMANOVA: pseudo-F = 13.49, R^2^ = 0.15, *P* < 0.01; Mann-Whitney tests, *P* < 0.05, Table 2) in all areas of interest. The AUC between VS/UWS and MCS was significant (confidence interval was higher than 0.5) for the following areas of interest: MVIS (AUC = 0.83; Bal ACCU = 0.65), LVIS (AUC = 0.78; Bal ACCU = 0.63), DAN (AUC = 0.77; Bal ACCU = 0.62), DMN (AUC = 0.78; Bal ACCU = 0.63), precuneus (AUC = 0.82; Bal ACCU = 0.66), and whole brain (AUC = 0.78; Bal ACCU = 0.62) (Table 2). Correlations of SUV values with CRS-R scores were significant for all areas (*ρ* range: 0.26–0.44), as well as with the CRS-R modified score (*ρ* range: 0.26–0.43) (Table 2). The highest correlations with the CRS-R score were observed for MVIS (*ρ*=0.43) and precuneus (*ρ*=0.44). Correlations with disease duration were not significant.

Considering the three etiologies separately, no significant results were observed (trend test, PERMANOVA, AUC/BAL ACCU, correlation with CRS-R and CRS-R modified score), except for vascular patients, who showed in the precuneus a significant correlation with CRS-R and CRS-R modified; Supplementary Table 3). For anoxic patients, in VS/UWS and MCS groups could not be compared because the MCS group included only 3 patients.

As shown in Fig. 4BCD, for the traumatic and vascular categories, patients in VS/UWS and MCS exhibit similar SUV values (*P* > 0.05). In contrast, anoxic patients in VS/UWS show markedly lower metabolism compared to patients in VS/UWS from other etiologies (PERMANOVA: pseudo-F = 25.14, R^2^ = 0.36, *P* < 0.01), thereby facilitating the distinction between VS/UWS and MCS at the group level.

Considering traumatic and vascular patients together (*N* = 55), only the trend test showed a significant gradual increase of metabolism in MVIS and precuneus (*P* < 0.05; Supplementary Table 3) while differences between VS/UWS and MCS were no longer significant (PERMANOVA *P* > 0.05) and even the AUC did not achieve significant results (the lower bound of the confidence interval was less than 0.5; Supplementary Table 3). Results on misclassified patients are reported in the Supplementary Materials. Correlations with CRS-R and CRS-R modified scores were observed in only a few areas of interest, with the highest correlations in precuneus (*ρ*=0.51; *ρ*=0.47) for both scores and MVIS (*ρ*=0.48) for CRS-R score (Table 2).

### Correspondence between SUV and identification of rs-fMRI networks

**Figure 5:**
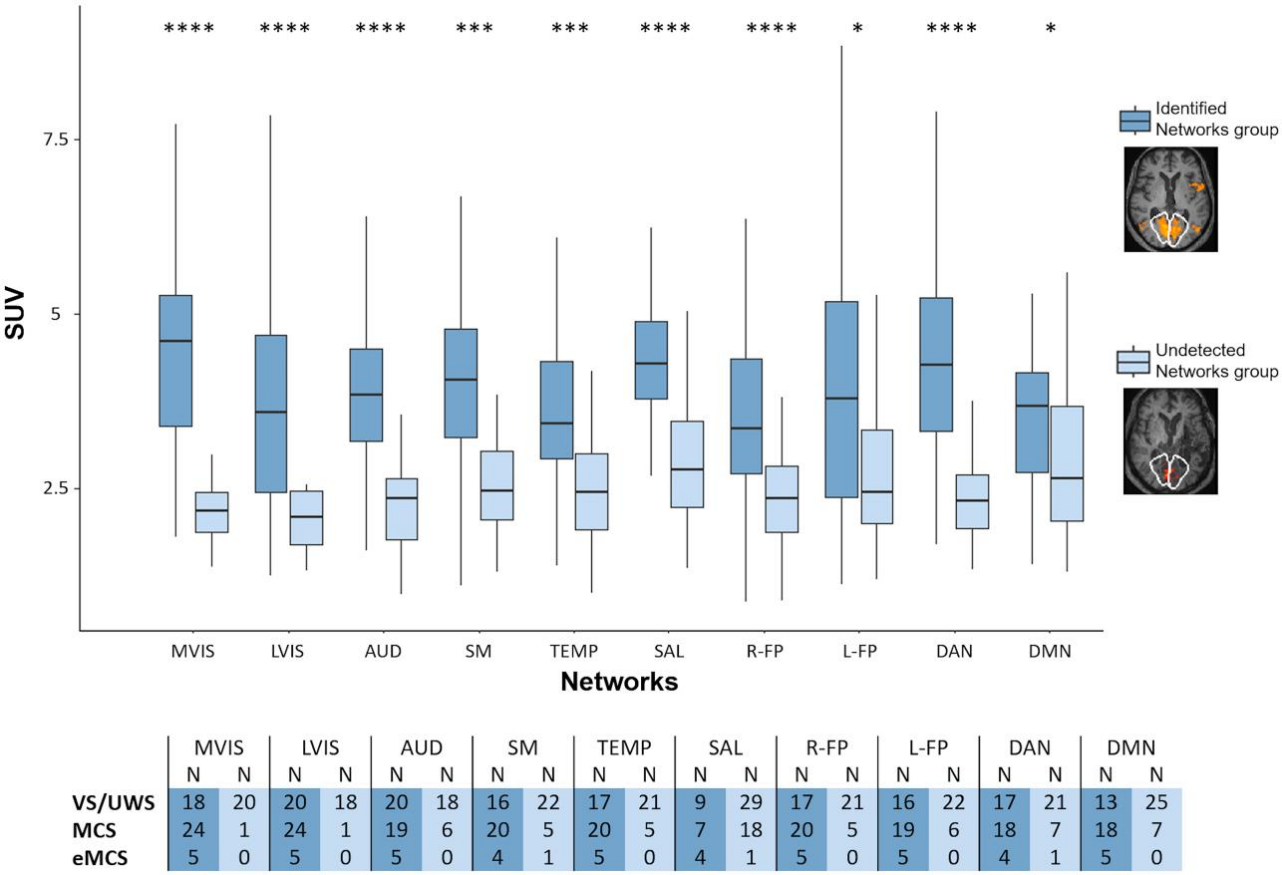
**SUV differences between the *Identified network* versus the *Undetected network* group based on rs-fMRI.** A boxplot of median SUV values for each network in the *Identified network* group and in the *Undetected network* group is reported. Differences between these two groups were assessed with the Mann-Whitney test, and *P*-values were reported, corrected for multiple comparisons using the Holm-Bonferroni method. The table below provides the corresponding numerosity of the *Identified* and *Undetected network* groups, differentiating by diagnosis (VS/UWS = 38, MCS = 25, and eMCS = 5). *N*, number. *P*-value: * < 0.05, ** < 0.01, *** < 0.001, **** < 0.0001.

Considering patients with both FDG-PET and rs-fMRI scans, the subsample includes 68 cases (38 VS/UWS, 25 MCS, and five eMCS). At a group-level, comparing patients showing a recognizable network (*Identified network* group) versus patients without recognizable networks (*Undetected network* group), the *Identified network* group displayed a higher SUV across all networks compared to the other group (Mann-Whitney test, *P* < 0.05, Fig. 5): this suggests that at the group-level, high FDG-PET values are associated with the identification of rs-fMRI network. The difference in metabolism varied from 22% to 49% across networks. However, across all networks, some individual patients showed networks despite low metabolic activity, resembling those in whom the network is undetected.

As reported in Table 3, after removing patients who had an MRI rating ≤ 1 in all seeds for each network, the comparisons between the *Identified network* versus *Undetected network* groups remained significant for only the SAL network (*P*  *<* 0.01). The results of the other networks are challenging to interpret due to the small size of the *Undetected network* group (see Table 3).

**Table 3:** Differences between identified and undetected network groups on rs-fMRI, excluding severely MRI-structurally damaged networks

Networks	*N* networks excluded (MRI rating ≤ 1)	*Identified network* group		*Undetected network* group		Mann-Whitney *U* test (*U, P-value*)
		*N*	SUV	*N*	SUV	
MVIS	18	47	4.64 (3.40–5.33)	3	2.38 (2.38–3.27)	28, 0.169
LVIS	16	49	3.59 (2.47–4.72)	3	4.26 (3.39–4.89)	90, 1.0
AUD	18	44	3.88 (3.18–4.63)	6	3.91 (2.62–4.48)	118, 1.0
SM	19	40	4.08 (3.34–4.83)	9	3.12 (3.00–5.29)	161, 1.0
TEMP	15	42	3.44 (2.94–4.59)	11	3.13 (2.39–3.90)	187, 1.0
SAL	19	20	4.28 (3.78–4.89)	29	3.12 (2.57–3.78)	126, *0.006*
R-FP	23	42	3.48 (2.73–4.32)	3	2.33 (2.04–2.84)	28, 0.732
L-FP	25	40	3.77 (2.37–5.18)	3	3.35 (2.59–4.53)	55, 1.0
DAN	18	39	4.38 (3.39–5.26)	11	2.43 (2.30–3.93)	125, 0.251
DMN	2	36	3.70 (2.78–4.12)	30	2.64 (1.96–3.79)	371, 0.235

Median SUV values (first and third quartiles) are reported. The difference between the *Identified network* group and the *Undetected network* group is presented for each network. All *P*-values are corrected for multiple comparisons using the Holm-Bonferroni method, and significant *P*-values are shown in italics. *N*, number of patients.

## Discussion

We investigated the metabolism of 84 chronic patients with DOC with different etiologies, assessing the diagnostic accuracy between VS/UWS and MCS in the whole group, as well as in the three etiological groups and also excluding the anoxic cases who had the lowest values. Then, the correspondence between FDG-PET and rs-fMRI was examined in a subgroup of 68 patients who underwent both techniques.

### Diagnostic accuracy of FDG-PET metabolism

In line with previous literature, our results confirm a specific pattern of increased metabolism as the clinical condition improved.^3,17,39-41^ As reported in the average SUV maps, patients in VS/UWS exhibited a widespread decrease in cortical metabolism compared to patients in MCS and eMCS, with preservation of activity mainly in the brainstem and cerebellum. By contrast, patients in MCS displayed a higher metabolism of bilateral posterior regions than other regions, including the precuneus, posterior cingulate, cuneus, lingual, fusiform, occipital gyri, caudate, and putamen. These regions showed significant differences when comparing VS/UWS versus MCS. In addition, patients in eMCS showed a higher metabolism overall than the other two diagnostic groups. This rising metabolism across diagnostic categories is confirmed by a trend analysis performed for each network and for the whole brain. Our results align with a well-known observation of increased metabolism specifically in the parieto-occipital areas,^4,39,42^ without observing any specific involvement of the thalamus and brainstem, at least as measured with FDG-PET.^3^ The increased metabolism in the striatum in MCS compared to VS/UWS patients is consistent with the mesocircuit hypothesis.^43^ Moreover, the distribution of metabolism in the CCP group appears to be intermediate between that of patients in VS/UWS and MCS, particularly in the posterior regions.

SUV values significantly correlate with CRS-R score (and CRS-R modified score) for all areas of interest, achieving a *ρ* of 0.43–0.44 for MVIS and precuneus, respectively. The correlations remain significant for the visual networks and the precuneus even when excluding the group of anoxic patients, reaching a *ρ*=0.51, similar to Hermann *et al*..^22^ By contrast, correlations with the disease duration are not significant as previously reported,^3,4^ likely due to the impact of various factors, including age and comorbidities associated with different etiologies.

Regarding diagnostic accuracy, FDG-PET metabolism could distinguish between patients in VS/UWS and in MCS, considering whole brain area as well as specific rs-fMRI networks. Specifically, SUV values in precuneus and MVIS yielded the highest diagnostic accuracy, achieving an AUC of 0.82–0.83 (Bal ACCU = 0.65–0.66), which is deemed very good based on the STARD guidelines.^44^ These were followed by LVIS, DMN, and DAN, reaching an AUC of approximately 0.77, considered to be good.^44^ The regions included in these networks (visual networks, DMN, and DAN) roughly correspond to the ‘maximum difference map’ of cerebral metabolic rate of glucose observed between patients in VS/UWS and in MCS (with an AUC of 0.87) in Stender *et al*..^3^

Our findings confirm the importance of specific regions like the precuneus^39^ and the DMN,^9,40^ and emphasize the significance of visual networks, including MVIS and LVIS, as supported by recent evidence.^10,11,45^ Nevertheless, considering a whole brain mask derived from EPI images, the AUC of the whole brain metabolism is still good (AUC = 0.78): this finding mirrors the results of previous studies that used values of cerebral metabolic rate of glucose of the cortex (AUC = 0.73)^3^ (although another index, the mean cortical metabolism in the best-preserved hemisphere, was reported even more effective at discriminating VS/UWS versus MCS (AUC = 0.82–0.89).^4,22^

### FDG-PET metabolism across etiological classes

Our results highlighted that the etiological class can profoundly affect the metabolic pattern of DOC. The causes leading to DOC typically are traumatic, vascular, and anoxic, with different pathophysiological mechanisms.^18,46^ In traumatic patients, injury stems from focal brain damage due to violent blows, with potential effects in distant areas for diffuse axonal injury.^47^ In vascular patients (haemorrhagic or ischemic stroke), damage is caused by a localized occlusion or rupture of vessels and is possibly associated with secondary damage such as reperfusion and neuroinflammation.^48^ In anoxic patients, the damage, typically due to cardiac arrest, results in reduced oxygen supply to the brain, leading to extensive injuries across various brain regions.^48^

The anoxic group (*N* = 29), consisting primarily of patients in VS/UWS (in line with other studies^18,22^), exhibited a pattern of pronounced hypometabolism. This widespread pattern^21,42,49^ was marked by reduced activity in the occipito-parietal areas, which are particularly vulnerable to injury under anoxic conditions.^49,50^ Their pattern differs significantly from that of traumatic and vascular patients, which is less severe and more variable depending on the site of damage.^42,48,51^

As reported above, in our group of 84 patients, the diagnostic accuracy between patients in VS/UWS and in MCS was very good (AUC = 0.82–0.83 in MVIS and precuneus). However, excluding anoxic patients, the diagnostic accuracy did not reach significance (the lower limit of the AUC confidence interval is less than 0.5) even though there remains a trend describing an increase in metabolism as the diagnosis improves for the MVIS and precuneus areas of interest (sample size: *N* = 55). Within the anoxic class, it was not possible to assess diagnostic differences since only three patients had an MCS diagnosis. This observation is in line with the high mortality rate observed specifically in anoxic patients.^52^

The literature on FDG-PET in DOC typically categorizes etiology into traumatic and non-traumatic, often without finding significant differences between the two groups,^3,4^ and therefore reports a single diagnostic accuracy value that can reach an excellent performance.^4^ This result might be associated with a high prevalence of anoxic patients in VS/UWS, where a significant hypometabolism accentuates the difference from patients in MCS. As reported by the European guidelines,^5^ FDG-PET demonstrates high diagnostic accuracy; however, future studies should assess the impact of various etiological classes on this accuracy.

### Correspondence between FDG-PET metabolism and rs-fMRI networks

In this study, we assessed the two functional techniques to understand their degree of correspondence^53^ in 68 patients (38 VS/UWS, 25 MCS, and five eMCS). The identification of rs-fMRI networks was associated with increased metabolism in the *Identified*  *network* group compared to the *Undetected*  *network* group in all networks, even when excluding patients with severe structural damage. A critical issue in rs-fMRI data analysis is the recognition of networks by separating the neuronal components from the artefactual ones.^54^ This is particularly relevant in DOC, where deformed brains can significantly alter the networks^17^ and the residual metabolism of severely damaged areas is less predictable. Indeed, the study by Soddu *et al*.^17^ which compared rs-fMRI with FDG-PET in patients with DOC, showed that while the similarity between the two techniques is high in healthy subjects (ρ=0.75), it is significantly decreased in patients in VS/UWS (ρ=0.58).

The results of our study show that the metabolism of patients with a recognizable network is significantly higher, up to 49%, compared to the metabolism of patients without recognizable networks. This difference is observed across all networks. In theory, this could be due to a high degree of anatomical damage, where patients without recognizable networks have greater underlying structural damage and, consequently, reduced metabolism. In practice, excluding the patients with the most structurally damaged nodes based on MRI data, the difference between patients with and without recognizable networks remains significant for the SAL. For the other networks, the number of patients was too imbalanced between the two groups to allow for a comparison. These results suggest that the identified neuronal networks are supported by a high residual metabolism. Indeed, previous studies report that changes in metabolic activity are closely tied to variations in functional connectivity,^17,55,56^ especially within visual regions^56,57^ and the DMN.^56,58^ This association is observed not only in the wakeful state^55,56^ but also during anaesthesia-induced unconsciousness.^56^

Considering the various diagnostic classes, our results on *Identified* versus *Undetected networks* confirm the correspondence between FDG-PET and rs-fMRI networks in patients in VS/UWS, as shown by Soddu *et al*.,^17^ and extend this observation to MCS and eMCS cases (Fig. 5). Our dataset does not include healthy control subjects undergoing FDG-PET, and we therefore lack a normative benchmark to define what constitutes absolute values of low versus high metabolism.^2^

In the attempt to translate research efforts into clinical practice,^59^ our study highlights that the presence of functional networks co-occurs with increased metabolic activity, thus providing group-level convergent evidence between the two techniques. Therefore, given the cost, invasiveness, and declining availability of FDG-PET in clinical settings, rs-fMRI may represent a concordant alternative for assessing residual brain function in patients with DOC.

### Limitations and future directions

Our study includes a number of limitations. First, in our sample, the anoxic group consisted of 24 VS/UWS and only three MCS: this unbalance prevented any kind of statistical analysis. Second, as reported above, our study lacks a healthy control group for comparison. Third, we have no longitudinal data that can shed light on the progression of the disorder, especially during its acute stages or in recovery. Finally, some results are not significant in the voxel-wise analysis but are significant in the ROI analysis (e.g. correlation analyses with CRS-R score), as the latter offers greater sensitivity to regional effects that may not survive strict correction. In the future, it will be important to investigate whether etiological differences are also observed in the acute phase or if they become more pronounced in the chronic phase.

## Conclusions

FDG-PET metabolism was investigated in 84 chronic patients with DOC and in association with rs-fMRI data in a subgroup of 68 cases. As clinical conditions improved among the groups, there was a consistent metabolic increase, with MVIS and precuneus providing the best diagnostic accuracy (AUC 0.83 and 0.82, respectively), followed by LVIS, DMN, and DAN. However, when anoxic patients were excluded, the diagnostic accuracy did not reach significance, although MVIS and precuneus retained a trend of gradual increase in metabolism as the diagnosis improved. At a group level, higher metabolism was associated with the identification of rs-fMRI networks, while lower metabolism was associated with undetected rs-fMRI networks, confirming a good agreement between FDG-PET and rs-fMRI. Among clinical variables, etiology can affect the diagnostic accuracy in chronic patients.

## Supplementary Material

fcaf495_Supplementary_Data

## Data Availability

Aggregated data are available upon request. Access to all group-level maps for patients in VS/UWS, MCS, CCP, and eMCS, also subdivided by etiology, at the link: https://neurovault.org/collections/21725/.
