# Supplementary material for: PET in conjunction with resting-state functional MRI for the study of chronic disorders of consciousness
Source: Brain Commun. 2025 Dec 23;8(1):fcaf495. doi: 10.1093/braincomms/fcaf495 (PMC12782017; doi:10.1093/braincomms/fcaf495)
Supplement: fcaf495_Supplementary_Data [file fcaf495_supplementary_data.pdf]

Supplementary Materials

PET in conjunction with resting-state functional MRI for the study of chronic disorders of consciousness

Methods:

The visual task used a block-design fMRI paradigm with house and face stimuli (details in Bertolino et al.[1]), while the auditory task was an event-related fMRI paradigm involving a hierarchical structure with pairs of pseudowords, unrelated words, and semantically related words - that is, stimuli differing in lexical status (words vs. pseudowords) and semantic relatedness (related vs. unrelated) (details in Nigri et al.[2]; Ferraro et al.[3]).

Supplementary Table 1. Subgroup of chronic VS and MCS- patients who underwent visual or auditory tasks for the assessment of covert cortical processing (CCP).

Patients	Diagnosis	Etiology	Disease duration	SUV value (whole brain)	Detected Networks	Visual task	Auditory task	CCP
pz_058	VS/UWS	Vascular	51	4,36	9	0	-	
pz_019	VS/UWS	Traumatic	48	3,98	8	1	-	1
pz_061	VS/UWS	Vascular	22	3,92	1	0	-	
pz_095	VS/UWS	Vascular	15	3,69	9	0	-	
pz_042	VS/UWS	Traumatic	25	3,62	9	0	-	
pz_132	VS/UWS	Traumatic	252	3,46	5	-	1	1
pz_068	VS/UWS	Vascular	9	3,41	10	0	-	
pz_013	VS/UWS	Vascular	23	3,37	9	0	-	
pz_030	VS/UWS	Anoxic	41	3,21	5	0	-	
pz_078	VS/UWS	Vascular	30	2,94	8	0	-	
pz_107	VS/UWS	Traumatic	9	2,90	9	1	-	1
pz_111	VS/UWS	Anoxic	23	2,82	-	0	-	
pz_041	VS/UWS	Traumatic	13	2,72	10	0	-	
pz_066	VS/UWS	Anoxic	42	2,64	0	0	-	
pz_129	VS/UWS	Vascular	12	2,48	9	0	-	
pz_098	VS/UWS	Vascular	9	2,31	4	0	-	
pz_031	VS/UWS	Anoxic	41	2,13	4	0	-	
pz_113	VS/UWS	Anoxic	14	2,07	0	0	-	
pz_131	VS/UWS	Anoxic	14	2,06	0	-	1	1
pz_124	VS/UWS	Anoxic	17	1,59	0	-	0	
pz_084	MCS -	Vascular	119	4,62	7	0	-	
pz_055	MCS -	Vascular	31	4,18	9	1	-	1
pz_143	MCS -	Traumatic	103	3,75	10	0	1	1
pz_126	MCS -	Vascular	15	3,59	3	-	0	
pz_054	MCS -	Vascular	29	2,97	4	0	-	
pz_036	MCS -	Vascular	37	2,90	9	0	-	
pz_104	MCS -	Traumatic	29	2,69	8	1	-	1
pz_059	MCS -	Vascular	20	1,69	4	1	-	1

A value of 1 in the CCP column indicates that the patient was classified as exhibiting covert cortical processing, based on the corresponding task (visual or auditory) in which a significant response in associative areas was detected. A dash (–) in the Visual task or Auditory task column indicates that the corresponding task was not administered. For reference, the average whole-brain SUV values for the diagnostic groups were VS/UWS = 2.86 ± 0.98 and MCS = 3.73 ± 1.21.

Results:

SUV in areas of interest: misclassified patients

Considering traumatic and vascular patients together, the AUC was not significant. There were 12 misclassified cases according to the AUC: four VS/UWS and eight MCS. The four VS/UWS had a higher number of recognizable networks compared to the remaining 19 VS/UWS correctly classified (8.75 ± 0.95 vs 6.72 ± 3.36, respectively), as well as high SUV values; on the other hand, the eight misclassified MCS cases had a lower number of recognizable networks compared to the 20 remaining MCS (6.71 ± 1.98 vs 7.33 ± 2.25, respectively), as well as low SUV values.

Supplementary Fig. 1. Color-blind accessible version of Fig. 1.

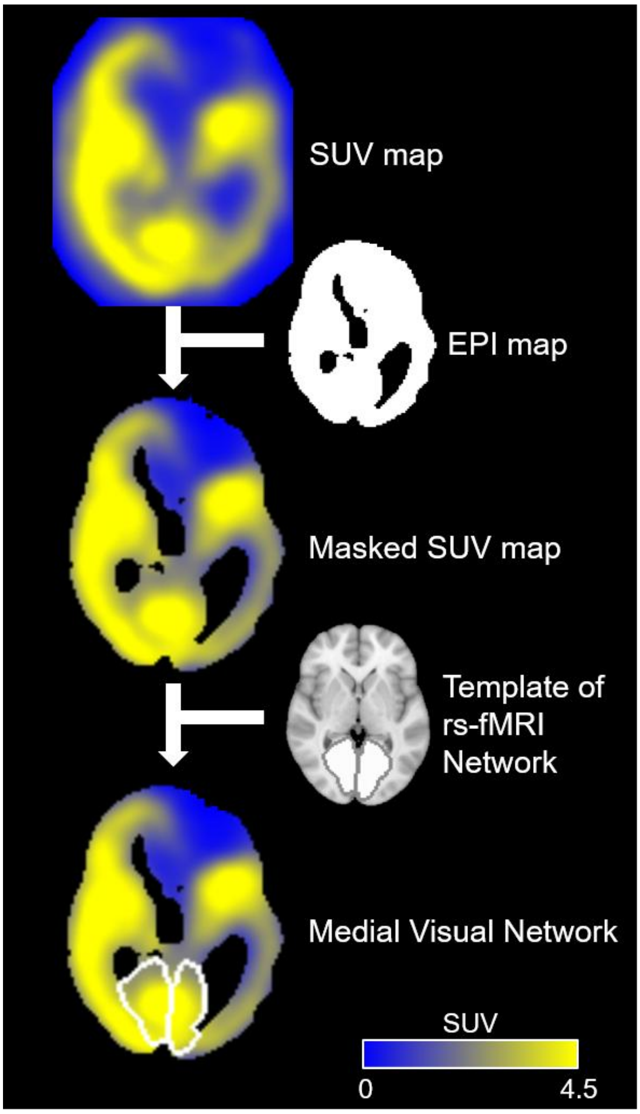

Workflow of the procedure applied to FDG-PET data of patients with DOC. The SUV map was masked using the EPI map to exclude areas of cerebrospinal fluid and anatomical damage. Then, a template for each area of interest (networks, precuneus, and whole brain) was used on the masked SUV map to extract SUV values. For illustrative purposes, a single subject is presented, and the MVIS template was superimposed.

Abbreviations: EPI, echoplanar images; SUV, standardized uptake values.

Supplementary Fig. 2. Color-blind accessible version of Fig. 2A.

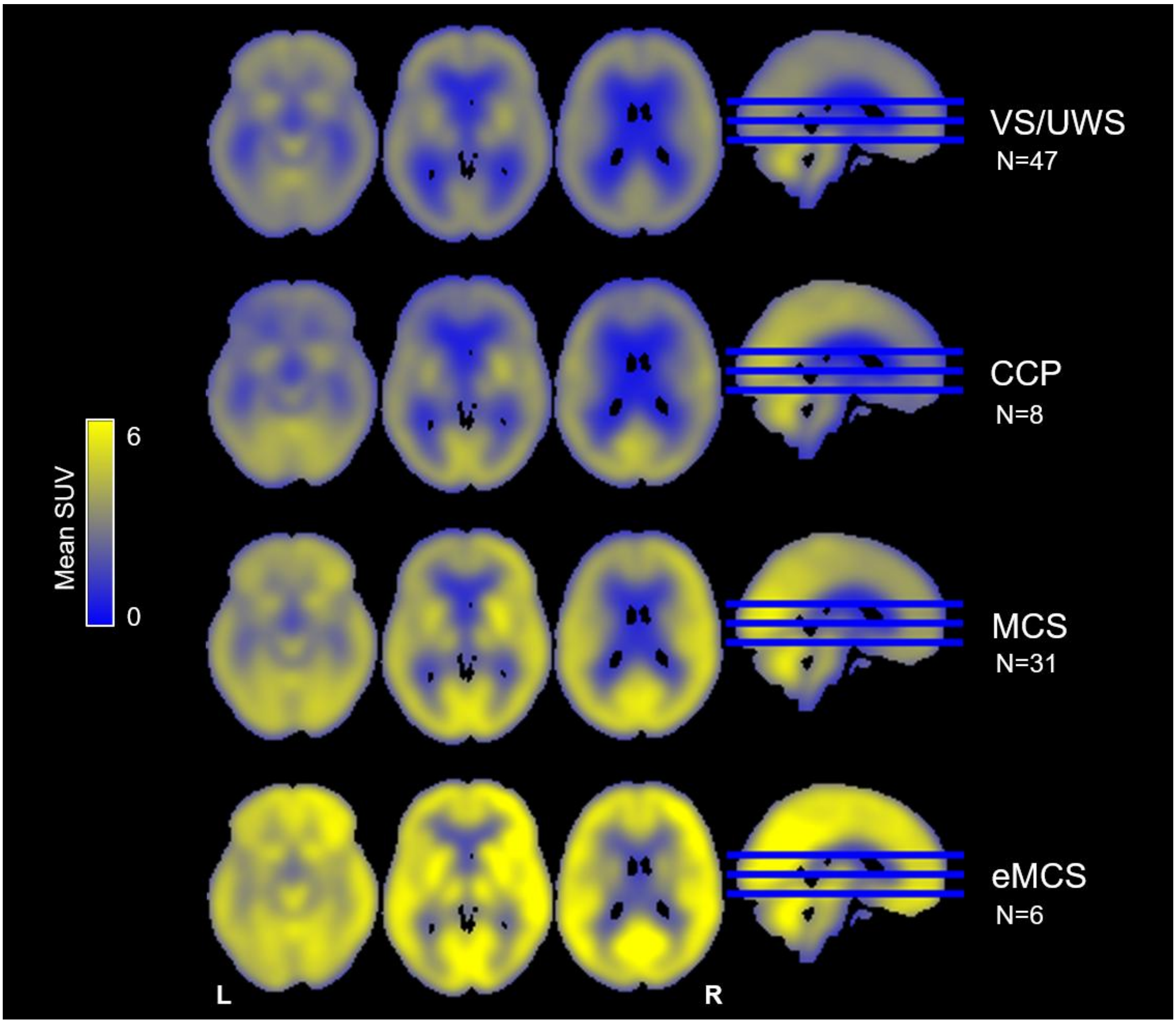

FDG-PET metabolism observed in patients in VS/UWS, MCS, and eMCS. A) Average maps of SUV values by diagnosis in VS/UWS ($N = 47$), CCP ($N = 8$), MCS ($N = 31$), and eMCS ($N = 6$) categories.

The axial sections are z = -14, 2, 18. Abbreviations: L, left; R, right.

Supplementary Fig. 3. Color-blind accessible version of Fig. 3.

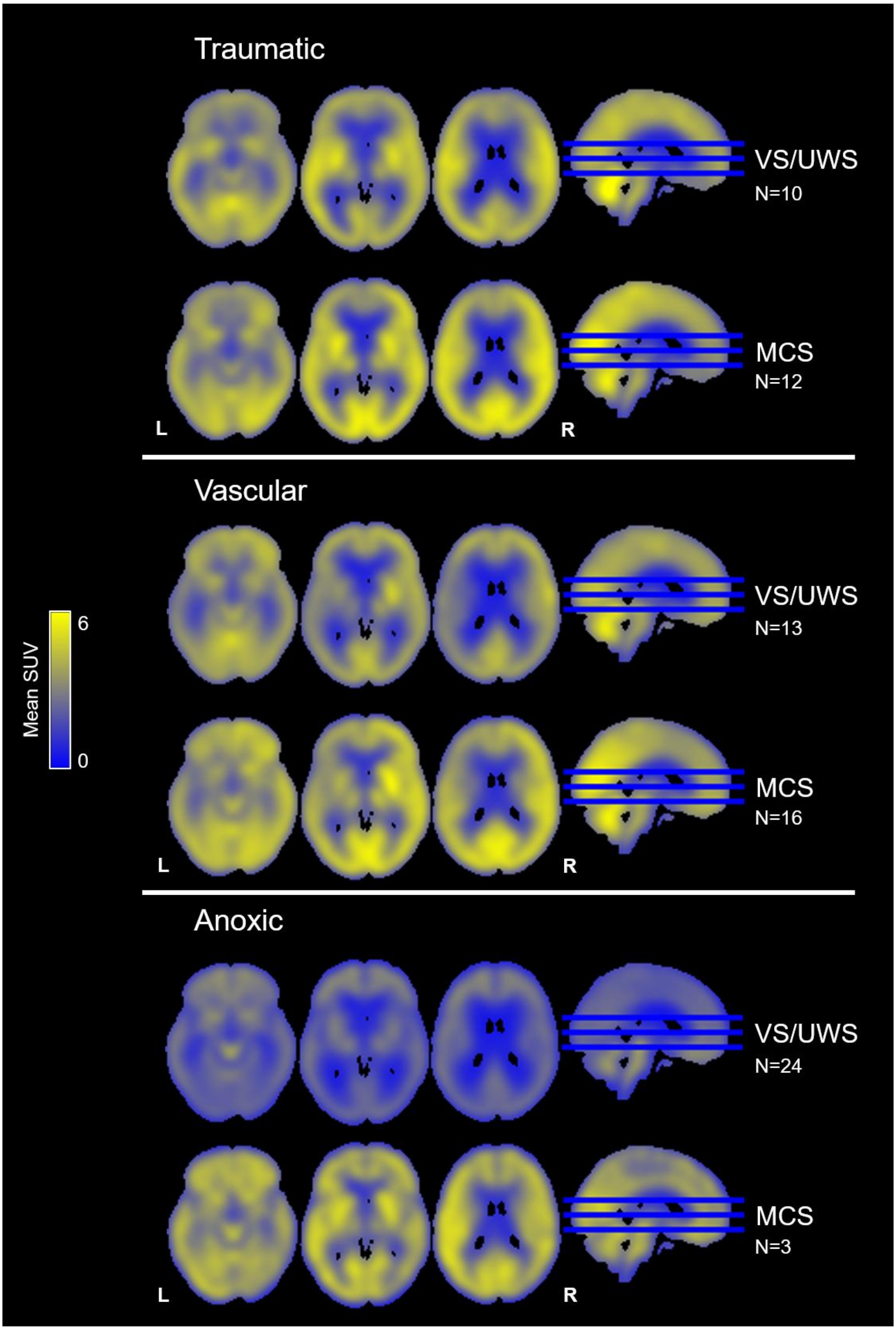

FDG-PET metabolism observed in patients in VS/UWS and in MCS grouped by etiology: traumatic, vascular, and anoxic. Average maps of SUV values by diagnosis for traumatic, vascular, and anoxic patients.

The axial sections are z = -14, 2, 18. Abbreviations: L, left; R, right.

Supplementary Fig. 4. T-score values in regions of significant difference between patients in VS/UWS and in MCS.

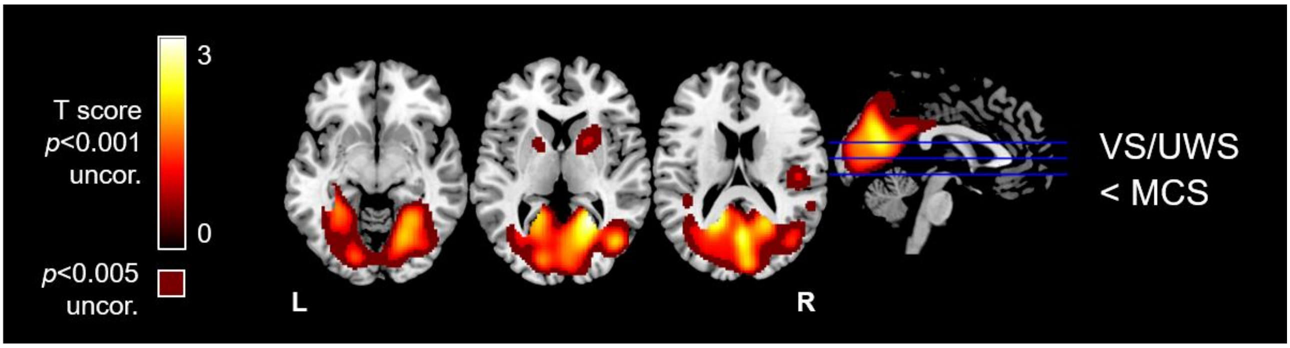

Regions of significantly higher metabolism in patients in MCS compared to patients in VS/UWS in the between-group voxel-wise analysis, using T-score with two thresholds of $p<0.001$ (red to yellow scale) and $p<0.005$ (dark red colour) uncorrected.

The axial sections are z = -8, 5, 18. Abbreviations: L, left; R, right; uncor., uncorrected.

Supplementary Table 2. Peaks of significantly higher metabolism in patients in MCS compared to patients in VS/UWS.

Cluster-level			Peak-level				Region
**N voxel**	$p_{FWE\text{-}corr}$	p_{uncorr}	**MNI coordinates**	***T-score***	$p_{FWE\text{-}corr}$	p_{uncorr}	
11791	**<0.001**	**<0.001**	22; -54; 6	4.77	**0.010**	**<0.001**	Calcarine R
			-18; -56; 14	4.64	**0.015**	**<0.001**	Calcarine L
			-12; -42; 28	4.56	**0.020**	**<0.001**	Posterior cingulate L
			2; -68; 22	4.51	**0.023**	**<0.001**	Precuneus R
			-12; -22; 36	4.39	**0.034**	**<0.001**	Middle cingulate L
			-12; -28; 34	4.33	**0.040**	**<0.001**	Middle cingulate L
			42; -70; 8	4.29	**0.045**	**<0.001**	Occipital middle R
137	0.38	0.56	26; 8; 10	3.43	0.39	**<0.001**	Putamen R
18	0.52	0.86	46; -20; 18	3.31	0.48	**<0.001**	Central operculum R

The results refer to the MCS > VS/UWS contrast reported in Fig. 2B and Supplementary Fig. 4. Significance levels are reported both uncorrected for multiple comparisons (uncorr) and corrected using family-wise error (FWE). Each peak MNI coordinate is associated with the corresponding brain region according to the AAL2 and Neuromorphometrics atlases.

Abbreviations: N, number; R, right; L, left.

Supplementary Table 3. SUV values results obtained for each area of interest (networks, precuneus, and whole brain) in traumatic, vascular, anoxic, and traumatic and vascular together etiologies.

Areas of Interest	Jonckheere-Terpstra trend test (*TJ, p-value*)	AUC [C.I.]	Bal ACCU	CRS-R (*ρ, p-value*)	CRS-R modified (*ρ, p-value*)
**Traumatic ($N = 24$)**					
	VS/UWS < MCS < eMCS	VS/UWS vs MCS		VS/UWS MCS eMCS	
MVIS	110, 0.75	0.73 [0.24-0.99]	0.60	0.47, 0.255	0.28, 1.0
LVIS	108, 0.75	0.72 [0.25-0.99]	0.59	0.40, 0.536	0.25, 1.0
AUD	97, 1.0	0.71 [0.22-0.99]	0.59	0.23, 1.0	0.03, 1.0
SM	97, 1.0	0.69 [0.22-0.99]	0.58	0.19, 1.0	0.03, 1.0
TEMP	95, 1.0	0.70 [0.21-0.99]	0.58	0.13, 1.0	-0.05, 1.0
SAL	103, 0.91	0.70 [0.22-0.99]	0.58	0.21, 1.0	0.16, 1.0
R-FP	94, 1.0	0.71 [0.25-0.99]	0.59	0.10, 1.0	0.02, 1.0
L-FP	103, 1.0	0.72 [0.25-0.99]	0.59	0.39, 0.557	0.25, 1.0
DAN	93, 1.0	0.68 [0.22-0.99]	0.58	0.20, 1.0	0.01, 1.0
DMN	105, 0.91	0.70 [0.27-0.99]	0.58	0.22, 1.0	0.11, 1.0
Precuneus	114, 0.65	0.72 [0.26-0.99]	0.59	0.44, 0.349	0.29, 1.0
Whole Brain	107, 0.81	0.71 [0.26-0.99]	0.59	0.31, 1.0	0.21, 1.0
**Vascular ($N = 31$)**					
	VS/UWS < MCS < eMCS	VS/UWS vs MCS		VS/UWS MCS eMCS	
MVIS	191, 0.13	0.74 [0.33-0.99]	0.60	0.46, 0.104	0.41, 0.269
LVIS	178, 0.45	0.70 [0.25-0.99]	0.58	0.34, 0.443	0.27, 0.913
AUD	156, 0.65	0.66 [0.20-0.99]	0.57	0.21, 0.774	0.23, 0.913
SM	160, 0.65	0.65 [0.19-0.99]	0.57	0.21, 0.774	0.15, 0.913
TEMP	171, 0.45	0.69 [0.26-0.99]	0.58	0.32, 0.468	0.36, 0.390
SAL	152, 0.65	0.65 [0.19-0.99]	0.57	0.27, 0.600	0.25, 0.913
R-FP	178, 0.45	0.70 [0.28-0.99]	0.59	0.41, 0.214	0.42, 0.241
L-FP	155, 0.65	0.65 [0.20-0.99]	0.57	0.15, 0.774	0.09, 0.913
DAN	185, 0.22	0.71 [0.29-0.99]	0.59	0.39, 0.252	0.37, 0.390
DMN	181, 0.23	0.70 [0.27-0.99]	0.58	0.40, 0.252	0.38, 0.387
Precuneus	200, 0.07	0.78 [0.41-0.99]	0.62	**0.51, 0.045**	**0.56, 0.021**
Whole Brain	173, 0.45	0.68 [0.25-0.99]	0.58	0.29, 0.600	0.27, 0.913

**Anoxic (*N* = 29)**					
	VS/UWS < MCS < eMCS	VS/UWS vs MCS		VS/UWS MCS eMCS	
MVIS	84, 0.472	-	-	0.03, 1.0	-0.06, 1.0
LVIS	65, 0.472	-	-	-0.09, 1.0	-0.16, 1.0
AUD	99, 0.336	-	-	0.24, 1.0	0.17, 1.0
SM	82, 0.472	-	-	0.04, 1.0	-0.02, 1.0
TEMP	94, 0.384	-	-	0.25, 1.0	0.17, 1.0
SAL	96, 0.374	-	-	0.15, 1.0	0.11, 1.0
R-FP	96, 0.374	-	-	0.17, 1.0	0.11, 1.0
L-FP	90, 0.396	-	-	0.21, 1.0	0.16, 1.0
DAN	86, 0.472	-	-	0.09, 1.0	-0.03, 1.0
DMN	94, 0.378	-	-	0.19, 1.0	0.10, 1.0
Precuneus	89, 0.420	-	-	0.09, 1.0	-0.08, 1.0
Whole Brain	91, 0.384	-	-	0.14, 1.0	0.12, 1.0
**Traumatic and Vascular (*N* = 55)**					
	VS/UWS < MCS < eMCS	VS/UWS vs MCS		VS/UWS MCS eMCS	
MVIS	593, **0.044**	0.70 [0.38-0.97]	0.59	0.48, **0.002**	0.39, 0.060
LVIS	561, 0.080	0.66 [0.33-0.96]	0.57	0.39, **0.037**	0.29, 0.418
AUD	508, 0.304	0.62 [0.27-0.95]	0.56	0.25, 0.252	0.17, 0.775
SM	516, 0.304	0.63 [0.28-0.95]	0.56	0.22, 0.252	0.14, 0.775
TEMP	522, 0.270	0.64 [0.28-0.96]	0.56	0.25, 0.252	0.20, 0.775
SAL	501, 0.304	0.62 [0.27-0.95]	0.55	0.24, 0.252	0.20, 0.775
R-FP	531, 0.270	0.65 [0.29-0.96]	0.57	0.29, 0.177	0.25, 0.554
L-FP	516, 0.304	0.62 [0.27-0.95]	0.55	0.29, 0.177	0.17, 0.775
DAN	539, 0.196	0.65 [0.31-0.96]	0.57	0.32, 0.130	0.24, 0.593
DMN	563, 0.081	0.66 [0.32-0.97]	0.58	0.34, 0.109	0.28, 0.435
Precuneus	614, **0.024**	0.72 [0.43-0.98]	0.60	0.51, **0.001**	0.47, **0.008**
Whole Brain	553, 0.152	0.65 [0.32-0.96]	0.57	0.33, 0.109	0.28, 0.435

From the top to the bottom sections are showed results of traumatic (*N* = 24), vascular (*N* = 31), anoxic (*N* = 29), and traumatic and vascular (*N* = 55) etiology samples. The trend test and correlations of SUV values with the CRS-R score and the modified CRS-R score were calculated considering the three diagnoses (VS/UWS, MCS, and eMCS). The AUC and Bal ACCU were obtained comparing patients in VS/UWS and in MCS. The PERMANOVA test did not show a significant global effect between patients in VS/UWS and in MCS in any etiological subgroup (Traumatic: pseudo-F = 0.59, $R^2 = 0.03$, $p = 0.56$; Vascular: pseudo-F = 1.71, $R^2 = 0.06$, $p = 0.19$; Anoxic: pseudo-F = 4.88, $R^2 = 0.16$, $p = 0.06$; Traumatic and Vascular: pseudo-F = 1.95, $R^2 = 0.04$, $p = 0.15$).

The results highlighted in bold indicate that the lower limit of the AUC confidence interval is higher than 0.5. All *p*-values are corrected for multiple comparisons using the Holm-Bonferroni method and significant *p*-values are shown in bold. Confidence intervals are reported at 95%. Abbreviations: C.I., confident interval.

References

1. Bertolino N, Ferraro S, Nigri A, Bruzzone MG, Ghielmetti F, The Coma Research Centre (CRC) multidisciplinary team, on behalf of which the present publication was submitted, acknowledges the following members: A Neural Network Approach to fMRI Binocular Visual Rivalry Task Analysis. Stamatakis EA, ed. *PLoS ONE*. 2014;9(8):e105206. doi:10.1371/journal.pone.0105206

2. Nigri A, Catricalà E, Ferraro S, et al. The neural correlates of lexical processing in disorders of consciousness. *Brain Imaging and Behav*. 2017;11(5):1526-1537. doi:10.1007/s11682-016-9613-7

3. Ferraro S, Nigri A, D'Incerti L, et al. Preservation of Language Processing and Auditory Performance in Patients With Disorders of Consciousness: A Multimodal Assessment. *Front Neurol*. 2020;11:526465. doi:10.3389/fneur.2020.526465

Data availability

The R code used for the statistical analyses is available at the following link:

https://irccsbesta-my.sharepoint.com/:f:/g/personal/alice_deruti_istituto-besta_it/Eu_i2tJ9yiVFvnoHHrbD7vsBVfki-Z6J2xKMqly_rzOqtQ?e=aAava9
